# 

**Published:** 1898-12

**Authors:** 


### INDEX TO VOLUME XIX.

A.

About Children, 133.
Abscessed Tooth, Death from an, 695.
Abstract of the Report of the Committee on Foreign Relations of the National Association of Dental Faculties, 713.
ABSTRACTS AND TRANSLATIONS:
- A New Factor in Erosion, 669.
- A Simple Saliva Injector, 801.
- Dentistry in Foreign Countries, 167.
- Four Cases of swallowing Artificial Teeth, 671.
- General Medical Council of Great Britain, 675.
- Gold Linings and Strengtheners for Vulcanite Plates, 724.
- Instrument Nomenclature with Reference to Instrumentation, 279.
- Iodoform Substitutes, 216.
- Liquefied Atmospheric Air, 220.
- New Theory in Regard to Caries of the Teeth, 673.
- Practical and Theoretical Trials of "Formagen," 358.
- Some Factors which cause Eruption of the Teeth, 94.
- The Administration of Anæsthetics, 514.
- The Preparation and Coloring of Lantern-Slides Suitable for Scientific Demonstrations, 15.
- The Relative Efficiency of Various Anæsthetics, 445.
- Why Coagulants diffuse through Dentine, 586.

Academy of Stomatology, 122, 230, 383, 534, 604, 678, 745.
Acquired Characteristics, The Transmission of, 561.
Act, A Questionable, 624.
Additional Dental Colleges not an Educational Necessity, 759.
Address, President's, 88.
Adhesive Cement for Plate- and Bridge-Work, 199.
Administration of Anæsthetics, 514.
Affections of the Mouth, Syphilitic, 162.
A Good Suggestion, 329.
Air, Liquefied Atmcspheric, 220.
Air, Liquid, 248.
Alcohol as a Disinfectant, 757.
Allan, George S., D.D.S. On Treatment of Pulpless Teeth with Hydrogen Dioxide, Ethereal Solution, 201.
Allan, George S., D.D.S. On Independent Journalism, 761.
Alloy, Bismuth in Dental, 693.
Alloy, Making Dental, 694.
Alumni Society of the Philadelphia Dental College, 491.
Amalgam, Gold in, 490.
Amalgam Question and Dentists with "Yankee Wit," The, 344.
Amalgam, Taveau's Pâte d'Argent not the First, 779.
Ambler, Henry L. On Tin-Foil and its Combinations for filling Teeth, 62.
American Academy of Dental Science, 48, 176, 362, 732.
American Academy of Dental Science, Resolutions of Respect, 196.
American and Southern Dental Associations, Joint Convention of, 45.
American Dental Association, 19, 101.
American Dental Association, The Funeral of the, 93.
American Dental Club of Paris, The, 134.
American Dentistry Abroad, 509.
American Medical Association, Section on Stomatology, 410.
A Method of crowning Broken-Down Teeth, 407.
A Move in the Wrong Direction, 549.
Anæsthesia, Local: Its Place in Surgery, 152.
Anæsthetics, 629.
Anæsthetics, The Administration of, 514.
Anæsthetics, The Relative Efficiency of Various, 445.
An Artificial Piece for an Ear, 277.
Anatomy, Descriptive and Surgical, 256.
An Epitome of the History of Medicine, 131.
A New Articulating Paper, 404.
A New Factor in Erosion, 669.
A Nice Legal Point,—Suppressing Irregular Medical Practice, 405.
Annual and Analytical Cyclopædia of Practical Medicine, 401.
Antidote for Arsenic Erosion, 694.
Antrum, Empyema of the, 496.
Antrum, Empyema of the, in an Infant, 622.
A Questionable Act, 624.
A Quiz Manual of Histology, General and Dental, 131.
A Reply from the New Jersey Secretary, 547.

A Reply to the Editor's Query, 403.
Arresting Hemorrhage, Novel Method of, 198, 261.
Arsenic Erosion, Antidote for, 694.
Arsenical Necrosis, 76.
Articulating Paper, A New, 404.
Articulation and Occlusion, 406.
Artificial Denture, The Preparation of the Mouth for an, preparatory to taking an Impression, 270.
Artificial Dentures in their Relation to the Speaking or the Singing Voice, 774.
Artificial Ear, Piece for an, 277.
Artistic Environment for the Dentist, 623.
Associate Lesions of the First Dentition, 641.
A Study of the Deformities of the Jaws among the Degenerate Classes of Europe, 1.
A System of Dental Surgery, 488.
A Text-Book of Dental Pathology and Therapeutics, including Pharmacology, 403, 550.
Atmospheric Air, Liquefied, 220.
A Treatise on Irregularities of the Teeth and their Correction, 195, 249.
A Treatise on Plateless Dentures, 691.

B.

Bad Methods, The Revival of, 247.
Barrett, W. C., M.D., D.D.S., M.D.S. On Oral Pathology and Practice, 612.
Bauchwitz, Max. On Practical and Theoretical Trials of "Formagen," 358.
Belcher, Harry L., D.D.S. Associate Lesions of the First Dentition, 641.
Bethel, L. P., D.D.S. Information for the Patient and Dentist, 819.
Bibliography:
About Children. By Samuel W. Kelly, M.D., 133.
Anatomy, Descriptive and Surgical. By Henry Gray, F.R.S., 256.
An Epitome of the History of Medicine. By Roswell Park, A.M., M.D., 131.
Annual and Analytical Cyclopædia of Practical Medicine. By Charles E. de M. Sajous, M.D., 401.
A Quiz Manual of Histology, General and Dental. By Charles B. Reed, M.D., 131.
A System of Dental Surgery. By the late Sir John Tomes, F.R.S., 488.
A Text-Book of Dental Pathology and Therapeutics, including Pharmacology. By Henry H. Burchard, M.D., D.D.S., 403, 550.
A Treatise on Irregularities of the Teeth and their Correction. By John Nutting Farrar, M.D., D.D.S., 195, 249.
A Treatise on Plateless Dentures. By C. A. Samsioe, 691.
Bibliography:
Cataphoresis or Electric Medicamental Diffusion as applied in Medicine, Surgery, and Dentistry. By William James Morton, M.D., 616.
Descriptive Anatomy of the Human Teeth. By G. V. Black, M.D., D.D.S., 402.
Elements of Latin. By George D. Crothers, A.M., M.D., 489.
Gould's Pocket Dictionary. By George M. Gould, M.D., 820.
Information for the Patient and Dentist. By L. P. Bethel, D.D.S., 819.
Oral Pathology and Practice. By W. C. Barrett, M.D., D.D.S., M.D.S., 612.
Orthodontia, or Malposition of the Human Teeth: Its Prevention and Remedy. By S. H. Guilford, A.M., D.D.S., 753.
Proceedings of the National School of Dental Technics, 257.
The American Dental Weekly. Edited by B. H. Catching, D.D.S., 257.
The Indiana Dental Journal. Edited by George Edwin Hunt, M.D., D.D.S., 194.
The Monthly Cyclopædia of Practical Medicine and Universal Medical Journal. Edited by Charles E. de M. Sajous, M.D., 256.
Tin-Foil and its Combinations for filling Teeth. By Henry L. Ambler, M.D., D.D.S., 62.
Transactions of the Illinois State Dental Society, 257.
Bismuth in Dental Alloy, 693.
Black, G. V., M.D., D.D.S. Descriptive Anatomy of the Human Teeth, 402.
Instrument Nomenclature with Reference to Instrumentation, 279.
Black's Theory, Dr., 756.
Blue Pencil, The, 198.
Board of Censors in Place of Examining Boards, 758.
Boice, Dr. Alonzo, 258.
Resolutions of Respect to, 557.
Bonwill, W. G. A., D.D.S. Reminiscences of a European Trip to the International Medical Congress at Moscow, Russia, August 19 to 26, 1897, 646, 707, 794.
Brackett, Charles A., D.M.D. The First Meeting with Patients, 582.
Bridge-Work, Adhesive Cement for Plate-and, 199.
Bridge-Work, Etruscan, 278.
Broken-Down Teeth, A Method of crowning, 407.
Bryan, L. C., D.D.S. On American Dentistry Abroad, 509.
Bull, Charles Stedman, A.M., M.D. On the Connection between Diseases of the Eye and Diseases of the Teeth, 137.
Burchard, H. H., M.D., D.D.S. On Anæsthetics, 629.

Burchard, H. H., M.D., D.D.S. A Text-Book of Dental Pathology and Therapeutics, including Pharmacology, 403, 550.
On Etruscan Bridge-Work, 278.
On some Reflex Disorders of Dental Origin, 697.
On Syphilitic Affections of the Mouth, 162.
Burned by Röntgen Rays, 198.
Burning by Röntgen Rays, 326.
Burns, Treatment for Carbolic Acid, 197.

C.

Calculus, Salivary, in the Parotid Gland, 758.
Calomel as a Curative Agent in Diphtheria, 259.
Carbolic Acid Burns, Treatment for, 197.
Caries of the Teeth, New Theory in Regard to, 673.
Cataphoresis or Electric Medicamental Diffusion as applied in Medicine, Surgery, and Dentistry, 616.
Catching, B. H., D.D.S., Editor *American Dental Weekly*, 257.
Central Dental Association of Northern New Jersey, 492.
Cervical Borders, Treatment of, 567.
Chance, George H., M.D., D.D.S., President's Address, 88.
Unite the Forces, 790.
Chapman, Dr. Albert Nelson, 327.
Character and Policy of Dental Journalism? What should be the, 426.
Characteristics, The Transmission of Acquired, 561.
Chase, Dr. Henry S., 258.
Chicago Dental Society, 492.
Chicago Dental Society,—Resolutions of Respect, 64.
Child, Replantation for, 562.
Children, About, 133.
Christian Science Helpful in Dental Practice, 756.
Clapp, Dwight M., D.M.D. On X-Rays, 86.
The First Meeting with Patients, 786.
Cleanly and Time-Saving Investment, 406.
Coagulants diffuse through Dentine, Why, 586.
Cocaine, Embarrassing Effects of, 329.
Cocainizing Pulps for Removal, 328.
Colorado State Dental Association, 412.
Colton, Dr. Gardner Quincy, 754.
Concerning Fees, 757.
Conditions that determine Choice of Filling-Materials, Physical, 503.
Connection between Diseases of the Eye and Diseases of the Teeth, The, 137.
Conventions, The Recent, 687.
Crothers, George D., A.M., M.D. Elements of Latin, 487.
Current News:
Alumni Society of the Philadelphia College, 491.
American Medical Association, Section on Stomatology, 410.
Central Dental Association of Northern New Jersey, 492.
Chicago Dental Society, 64, 492.
Colorado State Dental Association, 412.
Dental Society of the State of New York, 263, 492.
Harvard Dental Alumni Association, 564.
Harvard Odontological Society, 332.
Illinois State Dental Society, 264.
Iowa State Dental Society, 264.
Kansas State Dental Association, 264.
Massachusetts Dental Society, 330.
Meeting of the Vermont State Board of Dental Examiners, 628.
Missouri State Dental Association, 411.
National Association of Dental Examiners, 696.
National Association of Dental Faculties, 563.
National Dental Association, 409, 563.
National Dental Association, Division of the East, 331.
National School of Dental Technics, 826.
New Jersey State Dental Society, 410, 625.
New Jersey Summer Examinations, 411.
New York Odontological Society, Preliminary Notice, 825.
Ninth International Congress of Hygiene and Demography, 200.
Northeastern Dental Association, 696.
Pennsylvania State Board of Dental Examiners, 264, 824.
Pennsylvania State Dental Society, 412.
Rhode Island Dental Society, 200.
Southern Branch of the National Dental Association, Second Annual Meeting, New Orleans, La., February 9, 10, 11, and 13, 1899, 627.
Southern California Dental Association, 628, 759.
Tenth Anniversary of the Odontographic Society of Chicago, 136.
The New York Institute of Stomatology, 200.

D.

Davenport, S. E., D.D.S. Some Properties of Vulcanite Rubber, 579.
Davenport, W. S. On Spring Levers for regulating Teeth, 9.
Dawn of Modern Dental Literature, The, 333.
Death from an Abscessed Tooth, 695.
Deformities of the Jaw, A Study of, 1.
Degenerate Classes of Europe, A Study of the Deformities of the Jaws among the, 1.

Degrees, Report of Committee on Foreign, 750.
Demography, Ninth International Congress of Hygiene and, 200.
Dental Alloy, Bismuth in, 693.
Dental Alloy, Making, 694.
Dental Association, The National, 486.
Dental Journalism? What should be the Character and Policy of, 426.
Dental Literature, The Dawn of Modern, 333.
Dental Pulp, Removal of, 702.
Dental Society of the State of New York, 263, 492.
Dentine, Why Coagulants diffuse through, 586.
Dentist and Morals, The, 262.
Dentist, Artistic Environment for the, 623.
Dentistry Abroad, American, 509.
Dentistry in Foreign Countries, 167, 191.
Dentistry, Students practising, 405.
Dentistry, The Past and Present in, 207.
Dentistry? What is, 320.
Dentists, The Amalgam Question and, with "Yankee Wit," 344.
Dentition, Associate Lesions of the First, 641.
Denture, The Preparation of the Mouth for an Artificial, preparatory to taking an Impression, 270.
Descriptive Anatomy of the Human Teeth, 402.
Descriptive and Surgical Anatomy, 256.
Desecrators of the Grave, 623.
De Trey, Dr. Emile, 755.
Diphtheria, Calomel as a Curative Agent in, 259.
Diseases of the Eye, The Connection between Diseases of the Teeth and, 137.
Disinfectant, Alcohol as a, 757.
Disorders of Dental Origin, Some Reflex, 697.
Distinguishing Eucaine from Cocaine, Methods of, 623.
Divorcement of Dental Literature from Trade, The, 482.
Domestic Correspondence:
- A Reply to the Editor's Query, 403.
- Burning by Röntgen Rays, 326.
- Election of Delegates to National Association, 325.
- Legitimate Dental Practice hampered by Law, 821.
- Reply to Editorial, May, 1898, 558.
- What is Dentistry? 320.

Do the Nerves grow Old? 490.
Double Resection of the Lower Maxilla, 640.
Dr. Black's Review, 544.
Dr. Black's Theory, 756.
Dunn, William, D.D.S. On An Artificial Piece for an Ear, 277.
Dr. Dunning, The Method of using Soft Gold-Foil as practised and taught by, 211.

E.

Ear, An Artificial Piece for an, 277.
Editorial:
- A Move in the Wrong Direction, 549.
- A Reply from the New Jersey Secretary, 547.
- Dr. Black's Review, 544.
- General Medical Council of Great Britain, 690.
- Is Morality a Product of Law? 126.
- Liquid Air, 248.
- Practice of Dentistry in Foreign Countries, 191.
- Report of Committee on Foreign Degrees, 750.
- Special Notice, 819.
- Standard of Entrance raised, 320.
- The Divorcement of Dental Literature from Trade, 482.
- The Funeral of Dr. Thomas W. Evans, 487.
- The Future of Entrance Standards, 397.
- The Last Month of the Year, 817.
- The National Dental Association, 486.
- The National Dental Association, Division of the East, 317.
- The Need for Original Investigation, 214.
- The Recent Conventions, 687.
- The Recently Enacted Law of New Jersey, 317.
- The Relative Value of Technical Instruction, 243.
- The Revival of Bad Methods, 247.
- The Southern Branch of the National Dental Association, 246.
- The Tendency to lean on the Past, 609.
- Wrong Impressions, 60.

Editor's Query, A Reply to the, 403.
Educator, Nature as an, 197.
Election of Delegates to National Association, 325.
Elements of Latin, 489.
Embarrassing Effects of Cocaine, 329.
Empyema of the Antrum, 496.
Empyema of the Antrum in an Infant, 622.
Entrance Standards, The Future of, 397.
Epitome on the History of Medicine, An, 131.
Erosion, A New Factor in, 669.
Erosion, Antidote for Arsenic, 694.
Erosion, Mouth-Washes as the Cause of, 406.
Error in Practice, Serration an, 355.
Eruption of the Teeth, Some Factors which cause, 94.
Ethereal Solution, Treatment of Pulpless Teeth with Hydrogen Dioxide, 201.
Etruscan Bridge-Work, 278.
Evans, Dr. Thomas W., Funeral of, 487.
Evolution, Studies in, 206, 357.
Exposition, The Trans-Mississippi, 197.
Exudative Inflammation, The Philosophy of, 65.

F.

Factor in Erosion, A New, 669.
Factors which cause Eruption of the Teeth, Some, 94.
Farrar, John Nutting, M.D., D.D.S. On a Treatise on Irregularities of the Teeth and their Correction, 195, 249.
Fees, Concerning, 757.
Fillebrown, Dr. Thomas, Secondary Operation for Harelip, 565.
Filling-Materials, Physical Conditions that determine Choice of, 503.
First Dentition, Associate Lesions of the, 641.
First Meeting with Patients, The, 582, 786.
Foil, Method of using Soft, 265.
Forces, Unite the, 790.
Foreign Countries, Dentistry in, 167, 191.
Foreign Degrees, Report of Committee on, 750.
Foreign Restrictive Legislation, 199.
"Formagen," Practical and Theoretical Trials of, 358.
Formaldehyde in Solid Form, 15.
Four Cases of swallowing Artificial Teeth, 671.
Fracture of the Teeth, Restoration with Porcelain in, 493.
Fresh Air, not Violent Exercise, 408.
Funeral of Dr. Thomas W. Evans, The, 487.
Funeral of the American Dental Association, The, 93.
Future of Entrance Standards, 397.

G.

Gartrell, J. H. On Gold Linings and Strengtheners for Vulcanite Plates, 724.
Gelatine Substitute for Glass, 261.
General Medical Council of Great Britain, 675, 690, 695.
Geography of the Teeth, 260.
Glass, Gelatine Substitute for, 261.
Gold in Amalgam, 490.
Gold Linings and Strengtheners for Vulcanite Plates, 624.
Gould, George M., M.D. Pocket Pronouncing Dictionary, 820.
Granular Gum, Imitation, 262.
Grave, Desecrators of the, 623.
Gray, Henry, F.R.S. On Anatomy, Descriptive and Surgical, 256.
Guilford, S. H., A.M., D.D.S. Orthodontia, or Malposition of the Human Teeth: Its Prevention and Remedy, 753.
Gum, Imitation Granular, 262.

H.

Harelip, Secondary Operation for, 565.
Harvard Dental Alumni Association, 464.
Harvard Odontological Society, 332.
Hemorrhage, Novel Method of arresting, 198, 261.
History of Medicine, An Epitome on the, 131.
Hot Water to relieve Pain, 263.
Howe, J. Morgan, M.D.S., M.D. On Secrets and Patents *versus* Professional Progress, 413.
How to make One's Self Unpopular, 624.
Hubbard, Dwight L., M.D. On Artificial Dentures in their Relation to the Speaking or Singing Voice, 774.
Hunt, George Edwin, Editor *Indiana Dental Journal*, 194.
Hydrogen Dioxide, Ethereal Solution, Treatment of Pulpless Teeth with, 201.
Hygiene and Demography, Ninth International Congress of, 200.

I.

Illinois State Dental Society, 264.
Imitation Granular Gum, 262.
Impression, The Preparation of the Mouth for an Artificial Denture Preparatory to taking an, 270.
Impressions, Wrong, 60.
Independent Journalism, 433.
Independent Journalism from a Dental Stand-Point, 761.
*Indiana Dental Journal*, The, 194.
India Rubber and Iniquity, 408.
Infected Tooth-Root Canals, The Therapeutic Treatment of, 11.
Inflammation, The Philosophy of Exudative, 65.
Information for the Patient and Dentist, 819.
Inglis, Otto E., D.D.S. On Arsenical Necrosis, 76.
Iniquity, India Rubber and, 408.
Instrumentation, Instrument Nomenclature with Reference to, 279.
Instruments, What, shall I carry? 704.
International Medical Congress at Moscow, Russia, 646, 707.
Investigation, The Need for Original, 314.
Investment, Cleanly and Time-Saving, 406.
Iodoform Substitutes, 206.
Iowa State Dental Society, 264.
Irregularities of the Teeth and their Correction, A Treatise on, 195, 249.
Is Morality a Product of Law? 126.
Is the Use of Tobacco Injurious to the Teeth? 330.

J.

Jack, L. Foster, M.D., D.D.S. On Restoration with Porcelain in Fracture of the Teeth, 493.
Jack, Louis, D.D.S. On What should be the Character and Policy of Dental Journalism? 426.
Joint Convention of American and Southern Dental Association, 45.
Journalism, Independent, 433.
Journalism, Independent, from a Dental Stand-Point, 761.

K.

Kansas State Dental Association, 264.
Kelly, Samuel W., M.D., About Children, 133.
Kimball, Charles O., M.D. The Method of using Soft Gold-Foil as practised and taught by Dr. Dunning, 211.

L.

Legislation, Foreign, Restrictive, 199.
Legitimate Dental Practice hampered by Law, 821.
Liquefied Atmospheric Air, 220.
Liquid Air, 248.
Lesions of the First Dentition, Associate, 641.
Local Anæsthesia in Surgery, The Place of, 152.
Lord, Benjamin, D.D.S. On Method of using Soft Foil, 265.
Lower Maxilla, Double Resection of the, 640.
Luckie, S. Blair, D.D.S. On Physical Conditions that determine Choice of Filling-Materials, 503.
Lund, F. B., M.D. On the Place of Local Anæsthesia in Surgery, 152.

M.

Making Dental Alloy, 694.
Massachusetts Board of Registration in Dentistry,—Resolution of Respect, 195.
Massachusetts Dental Society, 330.
Maxilla, Double Resection of the Lower, 640.
Method of using Soft Foil, 265.
Methods of distinguishing Eucaine from Cocaine, 623.
Meeting with Patients, The First, 582.
Meeting of the Vermont State Board of Dental Examiners, 628.
Mills, Dr. G. Alden. On Patents *versus* No Patents, 667.
On the Funeral of the American Dental Association, 93.
Missouri State Dental Association, 411.
Modern Dental Literature, The Dawn of, 333.
Morality a Product of Law? Is, 126.
Morals, Professional Atmosphere and, 439.
Morals, The Dentist and, 262.
Morton, William James, M.D. Cataphoresis or Electric Medicamental Diffusion as applied in Medicine, Surgery, and Dentistry, 616.
Mouth, Syphilitic Affections of the, 162.
Mouth-Washes as the Cause of Erosion, 406.
Move in the Wrong Direction, 549.
Musson, Dr. Emma E. On Empyema of the Antrum, 496.

N.

National Association, Election of Delegates to, 325.
National Association of Dental Examiners, 696.
National Association of Dental Faculties, 563.
National Dental Association, 112, 409, 486, 563, 728, 804.
National Dental Association, Division of the East, 317, 331.
National Dental Association, The Southern Branch of the, 246.
National School of Dental Technics, 826.
Nature as an Educator, 197.
Necrosis, Arsenical, 76.
Nerves, Do they grow Old? 490.
Neurectomy for Tic Douloureux, 328.
New Articulating Paper, A, 404.
New Factor in Erosion, A, 669.
New Jersey State Dental Society, 410, 625.
New Jersey Summer Examinations, 411.
New Theory in Regard to Caries of the Teeth, 673.
New York Institute of Stomatology, The, 116, 184, 200, 222, 299, 448, 528, 591.
New York Odontological Society, 825.
Ninth International Congress of Hygiene and Demography, 200.
Nones, Robert H., D.D.S. On the Preparation of the Mouth for an Artificial Denture preparatory to taking an Impression, 270.
Northeastern Dental Association, 696.
Notes and Comments:
Additional Dental Colleges not an Educational Necessity, 759.
Adhesive Cement for Plate- and Bridge-Work, 199.
A Good Suggestion, 329.
Alcohol as a Disinfectant, 757.
A Method of crowning Broken-Down Teeth, 407.
A New Articulating Paper, 404.
Antidote for Arsenic Erosion, 694.
A Questionable Act, 624.
Articulation and Occlusion, 406.
Artistic Environment for the Dentist, 623.
Bismuth in Dental Alloy, 693.
Board of Censors in Place of Examining Boards, 758.
Burned by Röntgen Rays, 198.
Calomel as a Curative Agent in Diphtheria, 259.
Christian Science Helpful in Dental Practice, 756.
Cocainizing Pulps for Removal, 328.
Concerning Fees, 757.
Death from an Abscessed Tooth, 695.
Desecrators of the Grave, 623.
Do the Nerves grow Old? 490.
Dr. Black's Theory, 756.
Embarrassing Effects of Cocaine, 329.
Empyema of the Antrum in an Infant, 622.
Foreign Restrictive Legislation, 199.
Fresh Air, not Violent Exercise, 408.
Gelatine Substitute for Glass, 261.
General Medical Council of Great Britain, 695.
Geography of the Teeth, 260.

NOTES AND COMMENTS:
Gold in Amalgam, 490.
Hot Water to relieve Pain, 263.
How to make One's Self Unpopular, 624.
Imitation Granular Gum, 262.
India Rubber and Iniquity, 408.
Investment, Cleanly and Time-Saving, 406.
Iodine and Permanganate of Potassium Stains, 823.
Is the Use of Tobacco Injurious to the Teeth? 330.
Liquefied Air as a Beverage, 823.
Local Treatment for Pyorrhœa Alveolaris, 824.
Making Dental Alloy, 694.
Methods of distinguishing Eucaine from Cocaine, 623.
Nature as an Educator, 197.
Neurectomy for Tic Douloureux, 328.
Novel Method of arresting Hemorrhage, 198, 261.
Our Porcelain Teeth, 407.
Production and Use of Carborundum, 822.
Quick Flasking, 824.
Removal of Teeth from Rubber Plates, 490.
Replantation for Child, 562.
Salivary Calculus in the Parotid Gland, 758.
Student Societies, 562.
Students practising Dentistry, 405.
Suppressing Irregular Medical Practice, A Nice Legal Point, 405.
The Blue Pencil, 198.
The Dentist and Morals, 262.
The Remedies to use, 404.
The Transmission of Acquired Characteristics, 561.
The Trans-Mississippi Exposition, 197.
The Wave-Length of the Röntgen Rays, 562.
Tooth Extraction for Blindness, 823.
Tooth-Washes as the Cause of Erosion, 406.
To Restore Zinc for Casting, 694.
Treatment for Carbolic Acid Burns, 197.
Treatment of Root-Perforation, 262.
Novel Method of arresting Hemorrhage, 198, 261.

O.

OBITUARY:
American Dental Club of Paris, 134.
Boice, Dr. Alonzo, 258.
Resolutions of Respect to, 557.
Chapman, Dr. Albert Nelson, 327.
Chase, Dr. Henry S., 258.
Chicago Dental Society,—Resolutions of Respect, 64.
Colton, Dr. Gardner Quincy, 754.
De Trey, Dr. Emile, 755.
Pepper, Dr. William, 618.

OBITUARY:
Resolutions of Respect,—American Academy of Dental Science, 196.
Resolutions of Respect,—Massachusetts Board of Registration in Dentistry, 195.
Southwick, Dr. Alfred P., 621.
Occlusion, Articulation and, 406.
Odontographic Society of Chicago, The Tenth Anniversary of the, 136.
Odontological Society of Pennsylvania, 53, 389, 519.
Operation for Harelip, Secondary, 565.
Oral Pathology and Practice, 612.
ORIGINAL COMMUNICATIONS:
American Dentistry Abroad, 509.
Anæsthetics, 629.
An Artificial Piece for an Ear, 277.
Arsenical Necrosis, 76.
Artificial Dentures in their Relation to the Speaking or the Singing Voice, 774.
Associate Lesions of the First Dentition, 641.
A Study of the Deformities of the Jaws among the Degenerate Classes of Europe, 1.
Double Resection of the Lower Maxilla, 640.
Empyema of the Antrum, 496.
Etruscan Bridge-Work, 278.
Formaldehyde in Solid Form, 15.
Independent Journalism, 433.
Independent Journalism from a Dental Stand-Point, 761.
Method of Using Soft Foil, 265.
Patents *versus* No Patents, 667.
Physical Conditions that determine Choice of Filling-Materials, 503.
President's Address, 88.
Professional Atmosphere and Morals, 439.
Reminiscences of a European Trip to the International Medical Congress at Moscow, Russia, August 19 to 26, 1897, 646, 707, 794.
Removal of the Dental Pulp, 702.
Restoration with Porcelain in Fracture of the Teeth, 493.
Secondary Operation for Harelip, 565.
Secrets and Patents *versus* Professional Progress, 413.
Serration an Error in Practice, 355.
Some Properties of Vulcanite Rubber, 579.
Some Reflex Disorders of Dental Origin, 697.
Spring Levers for regulating Teeth, 9.
Studies in Evolution, 206, 357.
Syphilitic Affections of the Mouth, 162.
Taveau's Pâte d'Argent not the First Amalgam, 779.
The Amalgam Question and Dentists with "Yankee Wit," 344.
The Connection between Diseases of the Eye and Diseases of the Teeth, 137.

ORIGINAL COMMUNICATIONS:
The Dawn of Modern Dental Literature, 333.
The First Meeting with Patients, 582, 786.
The Funeral of the American Dental Association, 93.
The Method of using Soft Gold-Foil as practised and taught by Dr. Dunning, 211.
The Past and Present in Dentistry, 207.
The Philosophy of Exudative Inflammation, 65.
The Place of Local Anæsthesia in Surgery, 152.
The Preparation of the Mouth for an Artificial Denture preparatory to taking an Impression, 270.
The Therapeutic Treatment of Infected Tooth-Root Canals, 11.
Treatment of Cervical Borders, 567.
Treatment of Pulpless Teeth with Hydrogen Dioxide, Ethereal Solution, 201.
Unite the Forces, 790.
What Instruments shall I carry? 704.
What should be the Character and Policy of Dental Journalism? 426.
X-Rays, 86.
Original Investigation, The Need for, 314.
Orthodontia, or Malposition of the Human Teeth: Its Prevention and Remedy, 753.
Our Porcelain Teeth, 407.
Owen, John, M.B. On Four Cases of swallowing Artificial Teeth, 671.

P.

Pain, Hot Water to relieve, 263.
Park, Roswell, A.M., M.D. On an Epitome of the History of Medicine, 131.
Parotid Gland, Salivary Calculus in, 758.
Past and Present in Dentistry, The, 207.
Past, The Tendency to lean on the, 609.
Patents *versus* No Patents, 667.
Patents *versus* Professional Progress, Secrets and, 413.
Patients, The First Meeting with, 582, 786.
Peirce, C. N., D.D.S. On The Past and Present in Dentistry, 207.
Pencil, The Blue, 198.
Pennsylvania State Board of Dental Examiners, 264.
Pennsylvania State Dental Society, 412.
Pepper, Dr. William, 618.
Perry, Safford G., D.D.S. Treatment of Cervical Borders, 567.
Philosophy of Exudative Inflammation, The, 65.
Physical Conditions that determine Choice of Filling-Materials, 503.
Piece for an Ear, An Artificial, 277.
Plate- and Bridge-Work, Adhesive Cement for, 199.
Plates, Gold Linings and Strengtheners for Vulcanite, 724.
Porcelain Teeth, Our, 407.
Potter, William H., D.M.D. On Independent Journalism, 433.
Practical and Theoretical Trials of "Formagen," 358.
Practice of Dentistry in Foreign Countries, 191.
Practice, Serration an Error in, 355.
Practising Dentistry, Students, 405.
Preparation of the Mouth for an Artificial Denture preparatory to taking an Impression, The, 270.
Present in Dentistry, The Past and, 207.
President's Address, 88.
Proceedings of the National School of Dental Technics, 257.
Product of Law? Is Morality a, 126.
Professional Atmosphere and Morals, 439.
Professional Progress, Secrets and Patents *versus*, 413,
Properties of Vulcanite Rubber, Some, 579.
Pulp, Removal of the Dental, 702.
Pulpless Teeth, Treatment of, with Hydrogen Dioxide, Ethereal Solution, 201.
Pulps, Cocainizing, for Removal, 328.

Q.

Questionable Act, A, 624.

R.

Rays, Burned by Röntgen, 198.
Rays, X-, 86.
Recent Conventions, The, 687.
Recently Enacted Law of New Jersey, 317.
Reed, Charles B., M.D. A Quiz Manual of Histology, General and Dental, 131.
Reflex Disorders of Dental Origin, Some, 697.
Regulating Teeth, Spring Levers for, 9.
Relative Efficiency of Various Anæsthetics, The, 445.
Relative Value of Technical Instruction, The, 243.
Relieve Pain, Hot Water to, 263.
Remedies to use, 404.
Reminiscences of a European Trip to the International Medical Congress at Moscow, Russia, August 19 to 26, 1897, 646, 707, 794.
Removal of Teeth from Rubber Plates, 490.
Removal of the Dental Pulp, 702.
Replantation for Child, 562.
Reply from the New Jersey Secretary, 547.
Reply to Editorial, May, 1898, 558.
Report of Committee on Foreign Degrees, 750.
REPORTS OF SOCIETY MEETINGS:
Academy of Stomatology, 122, 230, 383, 534, 604, 678, 745.
American Academy of Dental Science, 48, 176, 362, 732.
American Dental Association, 19, 101.
Joint Convention of the American and Southern Dental Associations, 45.
National Dental Association, 112, 728, 804.

REPORTS OF SOCIETY MEETINGS:
Odontological Society of Pennsylvania, 53, 389, 519.
The New York Institute of Stomatology, 116, 184, 222, 299, 448, 528, 591.
Resection of the Lower Maxilla, Double, 640.
Resolutions of Respect, 64.
Resolutions of Respect,—American Academy of Dental Science, 196.
Resolutions of Respect,—Massachusetts Board of Registration in Dentistry, 195.
Resolutions of Respect to Dr. Alonzo Boice, 557.
Restoration with Porcelain in Fractures of the Teeth, 493.
Restore Zinc for Casting, To, 694.
Review, Dr. Black's, 544.
Revival of Bad Methods, The, 247.
Rhode Island Dental Society, 200.
Rogers, John, M.D. On the Philosophy of Exudative Inflammation, 65.
Rollins, William. On Formaldehyde in Solid Form, 15.
Röntgen Rays, Burned by, 198.
Röntgen Rays, Burning by, 326.
Röntgen Rays, The Wave-Length of, 562.
Root-Perforation, Treatment of, 262.
Rubber, India, and Iniquity, 408.
Rubber, Some Properties of Vulcanite, 579.

S.

Sajous, Charles E. de M., M.D. Annual and Analytical Cyclopædia of Practical Medicine, 401.
Editor of the *Monthly Cyclopædia of Practical Medicine and Universal Medical Journal*, 256.
Saliva Ejector, A Simple, 801.
Salivary Calculus in the Parotid Gland, 758.
Samsioe, C. A. A Treatise on Plateless Dentures, 691.
Sanders, J. J. H. On A Simple Saliva Ejector, 801.
Scientific Demonstrations, The Preparation and Coloring of Lantern-Slides Suitable for, 15.
Scott, J. Alfred. On the Preparation and Coloring of Lantern-Slides Suitable for Scientific Demonstrations, 15.
Secondary Operation for Harelip, 565.
Secrets and Patents *versus* Professional Progress, 413.
Serration and Error in Practice, 355.
Smith, B. Holly, M.D., D.D.S. On Professional Atmosphere and Morals, 439.
On Removal of the Dental Pulp, 712.
Societies, Student, 562.
Soft Foil, Method of using, 265.
Soft Gold-Foil, Method of using, as taught and practised by Dr. Dunning, 211.
Solid Form, Formaldehyde in, 15.
Some Factors which cause Eruption of the Teeth, 94.
Some Properties of Vulcanite Rubber, 579.
Some Reflex Disorders of Dental Origin, 697.
Southern Branch of the National Dental Association, Second Annual Meeting, New Orleans, La., February 9, 10, 11, and 13, 1899, 627.
Southern California Dental Association, 628, 759.
Southern Dental Association, Joint Convention of the American and, 45.
Southwick, Dr. Alfred P., 621.
Spring Levers for regulating Teeth, 9.
Standard of Entrance raised, 320.
Student Societies, 562.
Students practising Dentistry, 405.
Studies in Evolution, 206, 357.
Substitute for Glass, Gelatine, 261.
Substitutes, Iodoform, 206.
Suggestion, A Good, 329.
Suppressing Irregular Medical Practice, A Nice Legal Point, 405.
Surgery, The Place of Local Anæsthesia in, 152.
Syphilitic Affections of the Mouth, 162.

T.

Taft, Charles H., A.B., D.M.D., The Amalgam Question and Dentists with "Yankee Wit," 344.
Talbot, Eugene S., M.D., D.D.S. On a Study of the Deformities of the Jaws among the Degenerate Classes of Europe, 1.
On Double Resection of the Lower Maxilla, 640.
On Studies in Evolution, 206, 357.
Taveau's Pâte d'Argent not the First Amalgam, 779.
Technical Instruction, The Relative Value of, 243.
Technics, National School of Dental, 826.
Teeth, A Method of crowning Broken-Down, 407.
Teeth, A Treatise on the Irregularities of, and their Correction, 195, 249.
Teeth, Four Cases of swallowing Artificial, 671.
Teeth, Geography of, 260.
Teeth? Is the Use of Tobacco Injurious to the, 330.
Teeth, New Theory in Regard to Caries of the, 673.
Teeth, Our Porcelain, 407.
Teeth, Removal of, from Rubber Plates, 490.
Teeth, Restoration in Porcelain, in Fracture of the, 493.
Teeth, Some Factors which cause Eruption of the, 94.
Teeth, Spring Levers for regulating, 9.
Teeth, The Connection between Diseases of the Eye and Diseases of the, 137.
Teeth, Treatment of Pulpless, with Hydrogen Dioxide, Ethereal Solution, 201.
Tendency to lean on the Past, The, 609.
Tenth Anniversary of the Odontographic Society of Chicago, 136.

The Amalgam Question and Dentists with "Yankee Wit," 344.
The American Dental Club of Paris, 134.
The *American Dental Weekly*, 257.
The Administration of Anæsthetics, 514.
The Blue Pencil, 198.
The Connection between Diseases of the Eye and Diseases of the Teeth, 137.
The Dawn of Modern Dental Literature, 333.
The Dentist and Morals, 262.
The Divorcement of Dental Literature from Trade, 482.
The First Meeting with Patients, 582, 786.
The Funeral of Dr. Thomas W. Evans, 487.
The Funeral of the American Dental Association, 93.
The Future of Entrance Standards, 397.
The History of Medicine, An Epitome on, 131.
The *Indiana Dental Journal*, 194.
The Last Month of the Year, 817.
The Method of using Soft Gold-Foil as practised and taught by Dr. Dunning, 211.
The *Monthly Cyclopædia of Practical Medicine and Universal Medical Journal*, 256.
The National Dental Association, 112, 409, 486, 563, 728.
The National Dental Association, Division of the East, 317, 331.
The Need for Original Investigation, 314.
Theoretical Trial of "Formagen," Practical and, 358.
The Past and Present in Dentistry, 207.
The Philosophy of Exudative Inflammation, 65.
The Place of Local Anæsthesia in Surgery, 152.
The Preparation and Coloring of Lantern-Slides Suitable for Scientific Demonstrations, 15.
The Preparation of the Mouth for an Artificial Denture, preparatory to taking an Impression, 270.
The Recent Conventions, 687.
The Recently Enacted Law of New Jersey, 317.
The Relative Efficiency of Various Anæsthetics, 445.
The Relative Value of Technical Instruction, 243.
The Remedies to use, 404.
The Revival of Bad Methods, 247.
The Southern Branch of the National Dental Association, 246.
The Tendency to lean on the Past, 609.
The Therapeutic Treatment of Infected Tooth-Root Canals, 11.
The Transmission of Acquired Characteristics, 561.
The Trans-Mississippi Exposition, 197.
The Wave-Length of the Röntgen Rays, 562.
Tin-Foil and its Combinations for filling Teeth, 62.
Tobacco Injurious to the Teeth? Is the Use of, 330.
Tomes, Sir John, F.R.S., A System of Dental Surgery by the Late, 488.
Tooth, Death from an Abscessed, 695.
Tooth-Root Canals, The Therapeutic Treatment of Infected, 11.
To restore Zinc for Casting, 694.
Trade, The Divorcement of Dental Literature from, 482.
Transactions of the Illinois State Dental Society, 257.
Transmission of Acquired Characteristics, 561.
Treatment for Carbolic Acid Burns, 197.
Treatment of Cervical Borders, 567.
Treatment of Infected Tooth-Root Canals, The Therapeutic, 11.
Treatment of Pulpless Teeth with Hydrogen Dioxide, Ethereal Solution, 201.
Treatment of Root-Perforation, 262.
Trueman, William H., D.D.S. On The Dawn of Modern Dental Literature, 333.
Taveau's Pâte d'Argent not the First Amalgam, 779.

U.

Underwood, Arthur S., M.R.C.S., L.D.S. On A New Factor in Erosion, 669.
Unite the Forces, 790.

V.

Voice, Artificial Dentures in their Relation to the Speaking or the Singing, 774.
Vulcanite Plates, Gold Linings and Strengtheners for, 724.
Vulcanite Rubber, Some Properties of, 579.

W.

Warren, George W., DD.S. On The Therapeutic Treatment of Infected Tooth-Root Canals, 11.
What Instruments shall I carry? 704.
What is Dentistry? 320.
What should be the Character and Policy of Dental Journalism? 426.
Whittles, Dencer, L.D.S. On Some Factors which cause Eruption of the Teeth, 94.
Why Coagulants diffuse through Dentine, 586.
Worcester, J. W., M.D. On Serration an Error in Practice, 355.
Wrong Impressions, 60.

X.

X-Rays, 86.

Y.

"Yankee Wit," The Amalgam Question and Dentists with, 344.
York, E. Lawley, D.D.S. On Why Coagulants diffuse through Dentine, 586.

Z.

Zinc for Casting, To Restore, 694.

